# Psychosis developed on a travel to china – a case report

**DOI:** 10.1192/j.eurpsy.2021.2129

**Published:** 2021-08-13

**Authors:** A. Certo, E. Mendes

**Affiliations:** Department Of Psychiatry And Mental Health, Centro Hospitalar de Vila Nova de Gaia e Espinho, Vila Nova de Gaia, Portugal

**Keywords:** travel, psychosis, tourist

## Abstract

**Introduction:**

Psychosis it is a serious medical condition that could happen to anyone while travelling, even without a prior history of mental illness. Some psychotic episodes during travel likely are brief psychotic disorders. This is a poorly understood subject that seems to have an increasing incidence.

**Objectives:**

This work aims to present a clinical case of a patient who developed psychotic symptoms on her visit to China, and to provide a brief update review of this subject.

**Methods:**

We describe a case based on patient’s history and clinical data. We also searched and reviewed cases on “travel” AND “psychosis” and “tourist” AND “psychosis” using PubMed® database.

**Results:**

We report the case of a 41-year-old woman without psychiatric antecedents or substance use who developed psychotic symptoms during a travel to China. Symptoms resolved completely soon after returning to Portugal and admission to the psychiatric emergency service where an antipsychotic treatment was initiated. Psychosis in tourists typically occur in destinations with strong symbolic or mystical connotations and in individuals who travel alone for several days. The most common symptoms are hallucinations, delusions, ideas of reference and agitation. Most patients improved and returned to previous functioning.

**Conclusions:**

To improve the knowledge of travel-related psychosis it is important to identify the cases and the associated biological and clinical factors, later on it may be possible to identify the predictive factors of these psychosis. Further research are necessary to establish a possible association between brief psychotic episode and travel to China, as reports for tourists to Jerusalem and to Florence.

**Disclosure:**

No significant relationships.

